# Yeast Diversity during Spontaneous Fermentations and Oenological Characterisation of Indigenous *Saccharomyces cerevisiae* for Potential as Wine Starter Cultures

**DOI:** 10.3390/microorganisms10071455

**Published:** 2022-07-19

**Authors:** Yu Chen, Jiao Jiang, Yaoyao Song, Xiaomin Zang, Guoping Wang, Yingfang Pei, Yuyang Song, Yi Qin, Yanlin Liu

**Affiliations:** 1 College of Enology, Northwest A & F University, Yangling, Xianyang 712100, China; chenyu0718@nwafu.edu.cn (Y.C.); jiao.jiang@nwafu.edu.cn (J.J.); y-ysong@nwafu.edu.cn (Y.S.); xiaominzang@nwafu.edu.cn (X.Z.); wangguoping202206@163.com (G.W.); peiyingfang06@163.com (Y.P.); yuyangsong@nwsuaf.edu.cn (Y.S.); 2 Ningxia Helan Mountain’s East Foothill Wine Experiment and Demonstration Station of Northwest A&F University, Yongning 750104, China; 3 Shaanxi Engineering Research Center for Viti-Viniculture, Yangling, Xianyang 712100, China

**Keywords:** yeast diversity, spontaneous fermentation, *Saccharomyces cerevisiae*, wine, volatile compounds

## Abstract

Diversity of regional yeast can be influenced by geography, grape cultivars and the use of SO_2_, but at single vineyard scale in China, the impact of these factors on yeast population, particularly *Saccharomyces cerevisiae,* is not well studied. Here, we characterised yeast species and dynamics during spontaneous fermentations with/without SO_2_ using eight typical grape cultivars from Yuma vineyard in Ningxia wine region of China. Results show that distribution and abundance of yeast species varied by grape varieties, fermentation stage and SO_2_ treatment. A number of 290 *S. cerevisiae* isolates were further classified into 33 genotypes by Interdelta fingerprinting. A prevailing role of grape varieties in shaping the genetic divergence of *S. cerevisiae* in Yuma vineyard was observed, as compared to the impacts of fermentation stage and SO_2_ treatment. Pre-selected *S. cerevisiae* strains were subjected to vinification with Cabernet Sauvignon and Chardonnay. All strains completed fermentations but the physiochemical parameters and volatile profiles of wines were strain-specific. Some indigenous *S. cerevisiae* yielded more desirable aroma compounds compared to the commercial strains, among which NX16 and NX18 outcompeted others, therefore having potential for use as starters. This study provides comprehensive analysis on yeast diversity at vineyard scale in Ningxia. Information on the vinification using indigenous *S. cerevisiae* is of great value for improving Ningxia wine regionality.

## 1. Introduction

Indigenous yeast which exists naturally on grape vine tissues exerts great influences on modulating vine health, growth, and yields [1,2]. Complex and genetically divergent yeast is subsequently transferred to the grape must/juice, after which their population changes dynamically during the wine fermentation process [3]. Numerous yeasts present during fermentation play a crucial role in wine production through both alcoholic fermentation and the release of desirable secondary metabolites that potentially enhance the complexity of wine aroma [3,4]. Distinct wines with unique regionality can be driven by indigenous yeasts, highlighting the importance of these microbes in adding to the economic and cultural value of wine [5,6]. In fact, there is growing interest among winemakers in using indigenous yeasts that are better adapted to local grape varieties and winemaking conditions [7,8,9], thus reflecting the unique “microbial *terroir*” of a given region.

The distribution of autochthonous yeasts can be conditioned by various “*terroir*” elements, encompassing topography, grape varieties, climate, anthropogenic practices, abiotic stressors during fermentation, etc. [2,8,10,11]. Fermentative yeasts *Hanseniaspora*, *Pichia*, *Metschnikowia* and *Issatchenkia* are commonly found at initial stages of fermentation whilst *Saccharomyces cerevisiae* gradually dominates the microbial community as fermentation proceeds [12,13]. Whilst these yeasts all proffer conspicuous contributions to wine flavour and mouthfeel formation [14,15], the uninoculated fermentations are mainly completed by the diverse populations of indigenous *S. cerevisiae* due to its high tolerance to wine-associated stressors.

The biodiversity of regional *S. cerevisiae* and its impact on wine quality have been studied over decades, either at global or at individual strain level [11,16,17,18,19]. Many previous studies have observed intermediate to high levels of genetic divergence among autochthonous *S. cerevisiae* isolates [16,19,20,21], which is likely due to geographical attributes [22], the dynamic changes of the wine environment [23,24], and anthropogenic practices (e.g., SO_2_ treatment) [25,26]. For individual indigenous *S. cerevisiae* isolates, the production of secondary metabolites, including desirable volatile compounds, can be strain-dependent [16,18,27,28]. Recently, geographic differentiation of *S. cerevisiae* strains has been characterised at global [5] and regional scales [8,15,29], revealing the distribution of distinctive populations at geographically large scales. At smaller scales, several studies investigated the biodiversity of *S. cerevisiae* from different vineyards within the same region [30] or the same sub-region [21], and different sites within a single vineyard [3,31,32]. Significant genetic differences between *S. cerevisiae* isolates at smaller scales can be driven by geographic factors [21,30], in conjunction with grape varieties [3,21,31,32]. Additionally, Liu et al. [21] further suggested that the *S. cerevisiae* population can be the main driver of wine aroma profiles at sub-regional scale. Taken together, investigation of the genetic diversity of indigenous *S. cerevisiae* and evaluation of individual isolates for oenological traits are of high importance for selection of ideal strains that can enhance wine regional characteristics.

Ningxia, which is located at 37°43′–39°23′ N and 105°45′–106°47′ E, is a rising and rapidly developing wine region of China with ideal climate conditions for producing premium wines. A number of studies on evaluation of microbial diversity during spontaneous fermentations involved sampling sites from Ningxia [5,19,33], and these studies were conducted at country scale. However, at smaller scales, e.g., within a single vineyard in Ningxia, limited information is available on the biodiversity of yeasts, in particular *S. cerevisiae*, and how individual *S. cerevisiae* strains impact wine quality. Additionally, the effect of anthropogenic practices, including supplementation with SO_2_, on indigenous yeasts in this region requires further investigation. To address these questions, we sampled yeast communities during spontaneous fermentations with/without SO_2_ from eight typical cultivars in Yuma vineyard, Ningxia. Yeast colonies isolated during spontaneous fermentations were taxonomically identified using both culture-based and molecular identification approaches, and the *S. cerevisiae* isolates were differentiated using Interdelta fingerprinting. Further, the influence of grape varieties, fermentation stage and SO_2_ addition on yeast biodiversity and the evolution of yeast population was analysed. Finally, representative genotypes of *S. cerevisiae* were subjected to Cabernet Sauvignon and Chardonnay fermentations to evaluate their potential for industrial use as starter cultures.

## 2. Materials and Methods

### 2.1. Sampling

Eight *Vitis vinifera* (including Cinsault, Semillon, Riesling, Yan73, Cabernet Gernischet, Pinot Noir, Merlot, and Cabernet Sauvignon) from the vineyard of Yuma Wine Co., Ltd., Qingtong Xia, China, were chosen for this study. The vineyard is located at 106 08′ E, 38 02′ N, with an average of altitude of 1130 m, in Qingtongxia, Ningxia province, China. The mean distance between any two of the eight grape cultivars ranged from 50 to 500 m. The vineyard was commercially managed and the grapevines were managed using similar viticultural practices. Handpicked grapes were immediately destemmed, crushed (red grapes) and/or pressed (white grapes) prior to being loaded into clean and decontaminated 20 L tanks. Spontaneous fermentations were carried out in duplicate at Yuma Winery, without addition of any commercial yeast, following similar fermentation protocols. Apart from the SO_2_-free group, potassium metabisulfite (PMS) was added to the grapes at crush to yield approximately 40 mg/L total SO_2_ to investigate the effect of SO_2_ on indigenous yeast communities. Red grapes were held for 24 h at a cool temperature (known as cold-soaking) followed by warming the must to commence fermentation. Fermentation proceeded at 25 °C for red wines and 20 °C for white wines. Samples were collected for yeast population analysis at three stages: before fermentation, in the middle of fermentation (50% of residual sugar), and at the end of fermentation (residual sugar <4 g/L).

### 2.2. Yeasts Enumeration, Isolation and Molecular Identification

Enumeration of yeasts was performed as previously described [21]. Yeast colonies were isolated by spreading out the serially diluted fermentation samples on fresh Wallerstein Laboratory Nutrient (WLN) agar medium (ThermoFisher Scientific, USA). Yeast species were originally clustered based on the growth and morphological characters of colonies [34,35]. Typical strains were selected from each cluster for further molecular identification. DNA was extracted from pure colonies using BioFlux Yeast Genomic DNA Extraction Kit (ThermoFisher Scientific) following the manufacturer’s instructions. To confirm yeast species, the 26S rRNA D1/D2 domain was analysed with primers of NL1 (5′-GCATATCAATAAGCGGAAAAG-3′) and NL4 (5′-GGTCCGTGTTTCAAGACGG-3′). PCR amplification was conducted in 50 μL reactions using Mango Taq DNA polymerase (Bioline, Italy), and contained ~200 ng DNA template and 50 pmol of each primer. PCR was performed as follows: 95 °C for 5 min, followed by 36 amplification cycles of 94 °C for 1 min, 52 °C for 1 min, 72 °C for 80 s, and a final 10 min extension at 72 °C. PCR products were initially analysed by gel electrophoresis and purified using the Wizard^®^ SV Gel and PCR Clean-up System (*Promega*) prior to sequencing by Beijing Sunbiotech Co., Ltd, Beijing, China. Species identity was determined using the BLAST tool in NCBI (http://blast.ncbi.nlm.nih.gov/blast, accessed on 7 Decemeber 2021), considering an identity threshold of at least 98%.

### 2.3. Strain Typing of S. cerevisiae Isolates

Interdelta polymorphism fingerprinting was used to evaluate the genetic diversity in indigenous *S. cerevisiae* strains by PCR amplification using the delta 12 (5′-TCAACAATGGAATCCCAAC-3′) and delta 21 (5′-CATCTTAACACCGTATATGA-3′) primers. PCR reaction and amplification of genomic sequences were performed following Feng et al. [36]. The PCR products were separated on 2% w/v agarose gel stained with GelRed and visualised under UV light. The commercial strain Lalvin RC212 (Lallemand) which was extensively used by Yuma Winery, was applied as the reference to compare the Interdelta profiles of the indigenous *S. cerevisiae* isolates.

### 2.4. Fermentation Performance of Selected S. cerevisiae Strains

Appropriate genetic representatives were selected from the regional *S. cerevisiae* populations to evaluate their fermentation performance using Cabernet Sauvignon and Chardonnay grapes. A total amount of 40 mg/L SO_2_ was added to the grape must/juice in the form of PMS (80 mg/L) before the inoculation of selected indigenous *S. cerevisiae* strains at a rate of 1 × 10^6^ cells/mL in triplicate in 20 L tanks. The commercial wine yeast strains *S. cerevisiae* TXL (Lamothe-abiet, France) and XR (Laffort, France) were inoculated into Chardonnay juice and Cabernet Sauvignon must respectively, as control groups. For Cabernet Sauvignon fermentation, the grape must was cold-soaked for 24 h at a cool temperature before warming the must to start fermentation. Fermentation was performed at 20 °C with Chardonnay juice and 25 °C with Cabernet Sauvignon must. Samples were collected daily to monitor residual sugar until fermented to dryness (residual sugar less than 4 g/L). The resultant wines were centrifuged and stored for subsequent wine metabolites and volatile analysis. All fermentation process was carried out at Yuma Winery in 2019.

### 2.5. Profiling of Wine Composition

Residual sugar and ethanol were measured according to Chen et al. [37], and pH was determined by a pH meter. Titratable acidity and volatile acidity were evaluated following OIV-MA-INT-00-2020 [38]. Total and free SO_2_ were analysed as previously described [36].

Volatile compounds were analysed using head space-solid phase microextraction-gas chromatography with mass spectrometry (HS-SPME-GC-MS) following Lan et al. [39] with some modifications. In brief, 5.0 mL wine samples were added into a 15 mL glass vial containing 1.0 g NaCl and 10 μL internal standards (4-methyl-2-pentanol, 20 mg/L), and equilibrated at 40 °C with 400 rpm agitation for 30 min. A 50/30 μm DVB/CAR/PDMS SPME fiber (Supelco, Bellefonte, PA, USA) was immersed in the headspace for 30 min to extract volatiles with continuous heating and agitation at 250 rpm and 40 °C, and subsequently desorbed at 250 °C in the GC injector for 8 min. Analysis was performed using Agilent 6890 GC system coupled with an Agilent 5975 MS detector. Volatile compounds were separated on a HP-INNOWAX capillary column (60 m × 0.25 mm × 0.25 μm, J&W Scientific, Folsom, CA, USA). Volatiles were injected in splitless inlet mode and carried by helium at a constant flow rate of 1 mL/min. The initial temperature of the GC oven was set at 50 °C and held for 1 min, then increased to 220 °C by 3 °C/min, holding at this temperature for 5 min. The temperature of both the transfer line and the ion source was 230 °C whilst the quadrupole was set at 150 °C. The MS detector was operated in electron ionization mode at 70 eV and was scanned over a mass acquisition range of *m*/*z* 29–350 with a scan interval of 0.2 s.

Standard calibration curves were built using volatile compound standards in a synthetic wine medium (14% *v*/*v* ethanol, 5 g/L tartaric acid, pH 3.8). The standard mixture was blended with 10 μL internal standard (4-methyl-2-pentanol, 20 mg/L), and analysed according to the same HS-SPME-GC-MS protocol as described above. Agilent ChemStation was used to qualify and quantify the volatile compounds. The concentrations of compounds were calculated using calibration curves following Lan et al. [39].

The odour activity values (OAVs), which are commonly used to evaluate the contribution of volatile compounds to wine aroma profiles, were calculated by the following formula [39].

OAVs = VC/OTD

VC: Concentration of volatile compounds (μg/L);

OTD: Odour thresholds of volatile compounds (in wine). The OTD values reported by González-Álvarez et al. [40], Hu et al. [41], Welke et al. [42] and Xiao et al. [43] were used to calculate the OAVs for the volatile compounds detected in this study.

### 2.6. Statistical Analysis

A neighbour-joining (NJ) phylogenetic tree was generated by MEGA 7 software using the Maximum Likelihood methods. Unweighted Pair Group Method with Arithmetic mean (UPGMA) in Data Processing System (DPS) was analysed for the proximity relation of different *S. cerevisiae* strains. Analysis of Similarities (ANOSIM) was performed to determine the differences in *S. cerevisiae* genotype composition between designated partitions (grape varieties, fermentation process and SO_2_ addition) using PAST [44]. Data from yeast enumeration, chemical and volatiles analysis were subjected to one-way ANOVA analysis and were expressed as mean values ± standard deviations (SPSS Statistics, V17.0, IBM, USA). Principal components analysis (PCA) was performed using XLSTAT (Addinsoft SARL) to compare the wine fermented with different *S. cerevisiae* strains based on their volatile profiles.

## 3. Results and Discussion

### 3.1. General Yeast Population Profile

To evaluate yeast population profiles in spontaneous fermentations from grapes harvested within an individual vineyard, 48 duplicate samples covering eight grape varieties within the same vineyard, from the beginning, middle and end of fermentation were collected to analyse yeast population. Enumeration of yeast was performed using the traditional culture-dependent technique. Initially, yeast population was approximately 8 × 10^4^ cfu/mL when fermentation started. Yeast viability gradually increased as fermentation processed, with the maximum population being observed at the middle fermentation stage (5 × 10^7^ cfu/mL, Table S1). Following that, viability decreased, finally reaching approximately 7 × 10^6^ cfu/mL by the time fermentation terminated.

**Figure 1:**
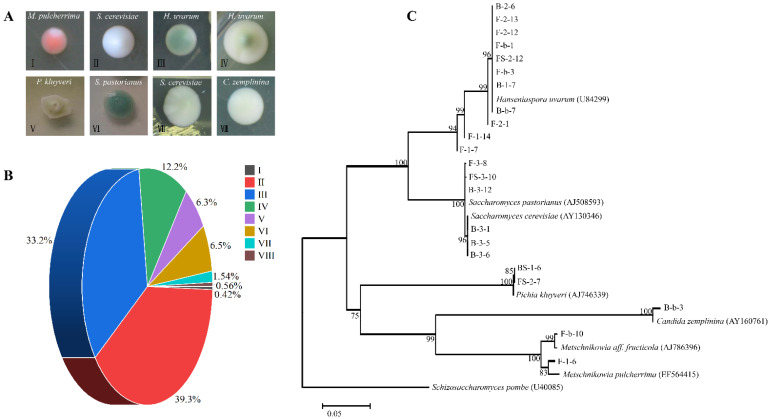
Species identification of 712 isolates obtained from spontaneous fermentations. (**A**): Colony morphotypes of eight yeast species on WLN agar medium. (**B**): Abundance of eight categories of morphotypes. (**C**): The neighbor-joining phylogenetic tree of selected isolates.

A total of 712 isolates were collected from three stages during spontaneous fermentation (Table S2). Based on colony morphology described by Li et al. [34] and Pallmann et al. [35], eight morphotypes were seen and six species were primarily identified (Figure 1A). Specifically, *Metschnikowia pulcherrima* (three colonies, category I), *S. cerevisiae* (290 colonies, category II, category VII), *Hanseniaspora uvarum* (323 colonies, category III, category IV), *Pichia kluyveri* (45 colonies, category V), *Saccharomyces pastorianus* (47 colonies, category VI), and *Candida zemplinina* (four colonies, category VIII) were identified among the 712 isolates (Table S3). The occurrence of these yeasts in spontaneous fermentations has been widely reported [8,37,45]. Notably, yeast isolates belonging to categories II and III were the most abundant, accounting for 39.33% (280 colonies) and 33.15% (236 colonies), respectively (Figure 1B). In contrast, isolates classified in categories I and VIII appeared sporadical, representing only 0.42% (three colonies) and 0.56% (four colonies) yeast presented spontaneous fermentation, respectively (Figure 1B).

A total of 22 representative strains selected from across all colony morphotypes were subjected to species identification by sequencing analysis of the taxonomically distinctive 26S rRNA D1/D2 domain (Table S3). To ensure accuracy, PCR amplification and sequencing were repeated multiple times. Molecular identification of 21 isolates at species level was identical with the outcomes gained from the WLN agar approach, highlighting the reliability of the traditional culture-dependent technique for preliminary wine yeast differentiation. Nonetheless, in only one case, we found that a *Metschnikowia aff. fructicola* strain F-b-10 verified by molecular species identification shared the same colony morphology as *M. pulcherrima* isolates (Figure 1C). Empirically, colony morphology can be similar among different species. Therefore, apart from using the conventional WLN agar approach, sequencing of the 26S rRNA gene [16] or the ITS regions of the ribosomal DNA [46] is still warranted for more accurate species identification. Further analysis on the phylogenetic relationship between the tested isolates was consistent with the morphotype clustering result (Figure 1C).

### 3.2. Yeast Population Dynamics during Spontaneous Fermentation

**Figure 2:**
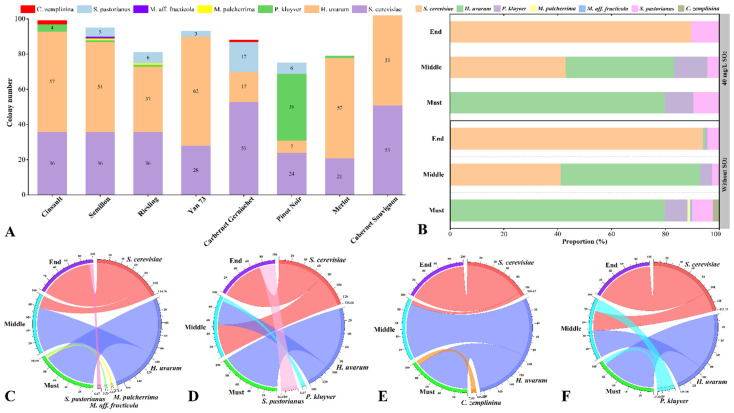
The distribution and dynamics of yeast during spontaneous fermentation. (**A**): Colony numbers of yeast species in spontaneous fermentations with different grape cultivars. (**B**): Relative abundance of yeast species during spontaneous fermentation with/without SO_2_ addition. The relative abundance of yeast species during SO_2_-free spontaneous fermentation with Semillon (**C**) and Cinsault (**E**). The relative abundance of yeast species during spontaneous fermentation with Semillon (**D**), Cinsault (**F**) treated with SO_2_. Must, before fermentation; Middle, the middle stage of fermentation; End, the end stage of fermentation.

The influence of grape variety and fermentation process on yeast population dynamics were analysed. Indigenous yeast exhibited distinct patterns of distribution across grape varieties in this particular vineyard (Figure 2A). While *S. cerevisiae* and *H. uvarum* were found from all fermentations, their abundance varied greatly between grape varieties. *P. kluyveri* dominated the yeast population in Pinot Noir fermentation whilst its presence was in low abundance with other fermentations (Figure 2A). *S. pastorianus* also possessed a wide distribution, with exception only in Cinsault, Merlot and Cabernet Sauvignon fermentations. Notably, albeit of low abundance, *M. pulcherrima* and *M. aff. fructicola* were only discovered in grape mash from Semillon and Riesling, respectively (Figure 2A), indicating their potential role in expressing distinct characteristics of wines made from the corresponding grape cultivar. In agreement with our study, several recent studies have also shown the role of grape varieties in differentiation of yeast populations during spontaneous fermentation [8,21]. Notedly, these studies revealed a picture of distinctive yeast populations with influence from grape varieties at regional (more than 100 Km; Gao et al. [8]) or sub-regional scales (8~12 km; Liu et al. [21]), whilst our study estimated the distribution of yeast patterns within an individual vineyard. Given the small scale of the studied area in our study, local conditions, including weather and topography may not be involved in modulating yeast communities between grape varieties. Factors like soil properties, harvest date, and animal vectors that can transfer yeast across the vineyard, (reviewed by Liu et al. [6]) might explain the intra-vineyard variation of yeast populations, but these analyses were beyond the scope of the current study.

Evolution of the yeast community was carefully evaluated throughout the fermentation (Figure 2B). At the start of fermentation, the grape must possessed higher species diversity, with *H. uvarum* dominating the yeast population (Figure 2B). Several previous studies also proved the high abundance of *H. uvarum* harboured in the grape juice/must [16,21], and discussed its role in improving wine aroma formation [41]. As fermentation proceeded, species diversity declined coinciding with increased population of *S. cerevisiae*, whose abundance finally reached 93.97% by the end of fermentation (Figure 2B). Decreased species diversity during fermentation can be due to the relatively poorer tolerance of many non-*Saccharomyces* to oenological associated stressors, in particular ethanol, compared to that of *S. cerevisiae* [47]. Nonetheless, non-*Saccharomyces H. uvarum* and *P. kluyveri* that have been reported as having average resistance to typical winemaking stressors [48] were seen throughout some fermentations (Figure 2B).

The effect of SO_2_ addition on the yeast community in spontaneous fermentation was also investigated in this study. Due to its antimicrobial function, SO_2_ is commonly used as an inexpensive and readily-implementable strategy to prevent the growth of undesirable microorganisms during winemaking. In this study, supplementation of SO_2_ before the onset of fermentation reduced yeast diversity during spontaneous fermentation, particularly at the beginning of fermentation (Figure 2B), which was in line with the results of Cureau et al. [49] and Morgan et al. [26]. Compared to the SO_2_-free group, SO_2_ addition resulted in the loss of some non-*Saccharomyces* yeasts when fermentation commenced, for example, *M. pulcherrima*, *M. aff. fructicola* in Semillon juice (Figure 2C,D) and *C. zemplinina* in Cinsault must (Figure 2E,F). These yeasts have been reported to be able to ferment at extremely low SO_2_ concentrations, offering paradigms for improving wine aroma complexity by releasing desirable secondary metabolites [50]. Therefore, to encourage the growth of the desirable non-*Saccharomyces* yeasts, and thereby enhance wine distinctiveness, SO_2_ usage should be carefully considered in a dose-dependent manner. Contrary to the suppressive impact on non-*Saccharomyces* yeast, SO_2_ treatment promoted the implantation of *S. cerevisiae* at middle fermentation points (Figure 2C–F). Considering the impact of fermentation process and anthropogenic practices, our work clearly showed that wine composition and SO_2_ treatment drive the microbial framework into a population dominated by *S. cerevisiae*, regardless of grape variety (Figure 2).

### 3.3. Genetic Diversity of S. cerevisiae by Interdelta Polymorphism Fingerprinting

**Figure 3:**
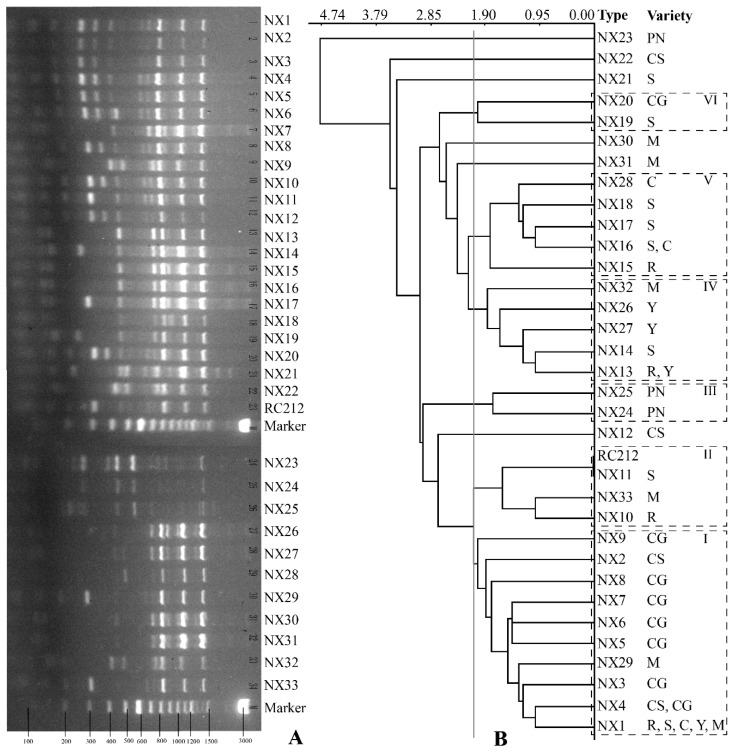
Interdelta fingerprinting of the indigenous *S. cerevisiae* clustering of isolates from Ningxia region. (**A**): Interdelta sequence profiles of the 33 distinctive genotypes. (**B**): UPGMA dendrogram generated from Interdelta fingerprinting patterns of 33 genotypes. C, Cinsault; CG, Cabernet Gernischet; CS, Cabernet Sauvignon; M, Merlot; PN, Pinot Noir; R, Riesling; S, Semillon; and Y, Yan73.

Since *S. cerevisiae* represented the most abundant species in spontaneous fermentation, the 290 indigenous *S. cerevisiae* isolates were subsequently analysed by Interdelta profiles to evaluate their genetic diversity. A total number of 33 distinct genotypes were obtained, and were coded NX1 to NX33 (Figure S1 and Figure 3A). Strains representing NX1 to NX9 genotypes had been isolated from the same vineyard from earlier vintages [36], indicating these indigenous *S. cerevisiae* strains might have successfully colonised the vineyard over vintages. Besides vintage scales, the genetic diversity of indigenous *S. cerevisiae* in vineyards can also be affected by the wide application of commercial starter cultures, which can be transferred from the winery either by insect vectors, or from liquid and solid winery deposits. Consequently, their subsequent sexual reproductive cycles would lead to the dispersal of commercial strains in the vineyard. Likewise, several studies have reported that a few *S. cerevisiae* strains isolated from spontaneous fermentations shared the same genetic pattern with commercial yeast strains commonly used in the corresponding viticultural region [51,52]. However, our results show that none of the Interdelta profiles of the 290 isolates were genetically related to that of Lalvin RC212, a commercial *S. cerevisiae* strain which has been extensively used in Yuma Winery (Figure S1). Comparison has further been made to the published Interdelta profiles of other commercial strains (EC1118 and Levuline SEWA) commonly used by Chinese winemakers [16]. Again, no colonisation of these commercial yeasts in the vineyard has been found (Figure S1). Taken together, our research outcome suggests that natural biodiversity of *S. cerevisiae* in Yuma vineyard may not be impacted by commercial starters, at least for the current single vintage. Future studies targeting indigenous *S. cerevisiae* population over consecutive years in Yuma vineyard are essential to elucidate the influence of commercial cultures.

Distribution of the *S. cerevisiae* genotype was strongly associated with grape variety, which was further confirmed by ANOSIM analysis (R = 0.5934, *p* = 0.0001). The most diverse genotypes were observed in fermentations with Merlot (9 types), followed by Semillon (8 types), and Cabernet Gernischet (7 types) (Table S4), indicating the adaptation of specific *S. cerevisiae* genotypes to the unique microhabitats formed by different grape cultivars. Generally, white wine fermentations harboured 11 distinct genotypes whilst the red wine fermentations contained 25 different genotypes (Table 1). NX10, NX11, NX14, NX15, NX17, NX18, NX19, and NX21 were only specific to white wine fermentations, while 22 genotypes were only found in red wine fermentations (Table 2). Particular attention should be paid to NX1, which was the most frequently encountered genotype in a wide range of spontaneous fermentations (except Cabernet Sauvignon, Cabernet Gernischet and Pinot Noir). Aside from NX1, albeit less frequent, NX13 and NX16 were also observed in both white and red wine fermentations. Given that the distance between any two of the grape varieties was within 500 m, insect vectors are likely to homogenise the yeast populations [53], and this might explain why NX1, NX13 and NX16 were observed in fermentations with multiple grape varieties.

The impact of fermentation progress on the distribution of *S. cerevisiae* populations was further analysed. NX4 and NX20 were the only two biotypes found from the grape must whilst a number of 15 and 18 genotypes were encountered at the middle and end of fermentation, respectively (Table 1). Among those genotypes seen at the middle of fermentation, 10 genotypes, including NX1, NX2, NX3, NX4, NX5, NX12, NX13, NX16, NX23 and NX27, could also be found towards the end of fermentation (Table 1). In contrast, NX8, NX10, NX14, NX21 and NX24 were only detected at the middle stage (Table 1). Nonetheless, ANOSIM analysis did not show any significant genetic differentiation of the temporal distribution of *S. cerevisiae* genotypes (R = −0.07591, *p* = 0.8964).

Genetic divergence of indigenous *S. cerevisiae* can also be weakly but not significantly altered by SO_2_ treatment, further confirmed by ANOSIM analysis (R = −0.02188, *p* = 0.6857). The number of *S. cerevisiae* genotypes in most SO_2_-free groups were reduced compared to the corresponding SO_2_-added groups (Table S4). Notably, some novel genotypes were seen in SO_2_-treated fermentations compared to the SO_2_-free groups, for example, NX18 and NX33 (Table 1), suggesting that SO_2_ addition may impact the evolution of *S. cerevisiae* during the course of fermentation.

To further investigate the clonal relationships between genotypes, the Interdelta data was used to build a dendrogram from the Euclidean distance, shown in Figure 3B. A total number of 12 groups were identified from the 33 genotypes at 2.22 Euclidean distance, among which six major groups (I–VI) was composed of at least two genotypes. The dendrogram showed that clustering of the genotypes was influenced by grape varieties (Figure 3B). Group I gathered 10 genotypes mainly originated from Cabernet Gernischet fermentations whilst Group III clustered genotypes solely isolated from Pinot Noir fermentation. Further investigation is required to determine whether these genotypes can be considered as potential microbial signatures of local Cabernet Gernischet and Pinot Noir wines [31]. Groups II, IV, V and VI harboured genotypes from two or more grape varieties.

### 3.4. Vinification Using Indigenous S. cerevisiae Strains with Differentiated Genotypes

It has been reported that the genotype of *S. cerevisiae* has a crucial impact on wine characteristics [54]. Here, to investigate how genetic differences of indigenous *S. cerevisiae* influence vinification profiles, 14 strains with varying genotypes were subjected to fermentations with Cabernet Sauvignon and Chardonnay. Nine representative genotypes found from uninoculated red grape fermentations (NX2, NX2, NX3, NX4, NX5, NX12, NX22, NX23 and NX24) (Table 1) were inoculated into Cabernet Sauvignon must whilst NX15 and NX21 obtained from white grape spontaneous fermentations (Table 1) were evaluated with Chardonnay juice. NX1, NX13 and NX16 commonly seen in spontaneous fermentations with multiple grape varieties (Table 1) were tested in both Cabernet Sauvignon and Chardonnay fermentations. All tested strains fermented to dryness (residual sugar < 4 g/L), and had little to no impact on alcoholic fermentation duration in any juice/must.

**Figure 4:**
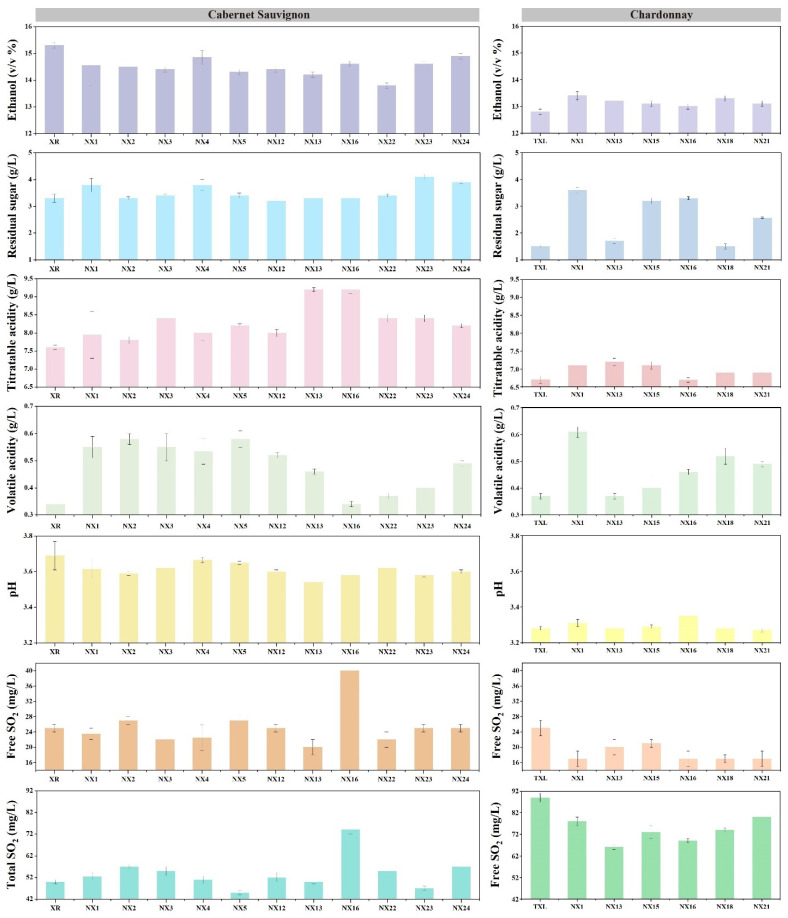
Physiochemical parameters of Cabernet Sauvignon and Chardonnay wines fermented by indigenous *S. cerevisiae*.

The resultant wines were analysed for ethanol, pH, titratable acidity (TA), volatile acidity (VA), free SO_2_ and total SO_2_. All parameters were within the acceptable ranges referring the National Standard of the People’s Republic of China (GB/T 15038-2006); however, they varied by genotypes (Figure 4). For instance, NX1 yielded higher amount of ethanol but low to intermediate TA (Figure 4). In contrast, NX13 constantly yielded the highest level of TA and decreased ethanol, suggesting partial diversion of carbon from ethanol production to organic acids. Acidification traits of indigenous *S. cerevisiae* can be affected by the interaction of grape varieties, e.g., NX16 resulted in high TA in Cabernet Sauvignon wines whilst yielding a significantly lower TA in Chardonnay wines (Figure 4). Empirically, *S. cerevisiae* strains would not lead to large variations in TA or pH after alcoholic fermentation. Microbial solutions to wine acidification are more commonly associated with the application of non-*Saccharomyces*, e.g., *Lachancea thermotolerans* [55]. Nonetheless, Feng et al. [16] had reported that several indigenous *S. cerevisiae* strains obtained from Qilian vineyards, China, were capable of producing significantly higher total acidity compared to the commercial strain EC1118. This acidifying trait has particular oenological significance since increasingly common inadequate acidity found in high sugar must/juice can be corrected by inoculation with such *S. cerevisiae* strains.

**Figure 5:**
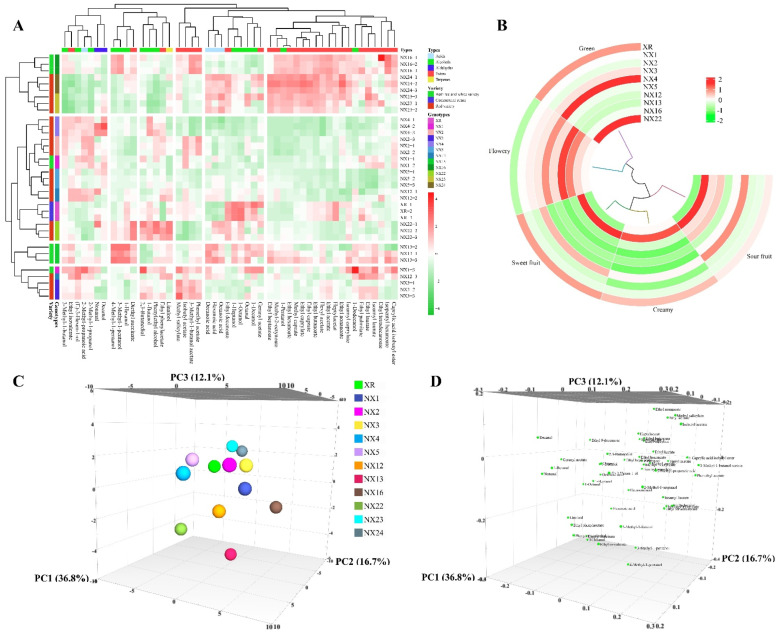
Volatile profile in Cabernet Sauvignon wines fermented by 11 indigenous *S. cerevisiae* strains with varying genotypes. (**A**): Heatmap analysis of volatile compounds in Cabernet Sauvignon wines. (**B**): Aroma attributes of the Cabernet Sauvignon wines. Principal component analysis (PCA) score (**C**) and loading (**D**) plots of the Cabernet Sauvignon wines.

**Figure 6:**
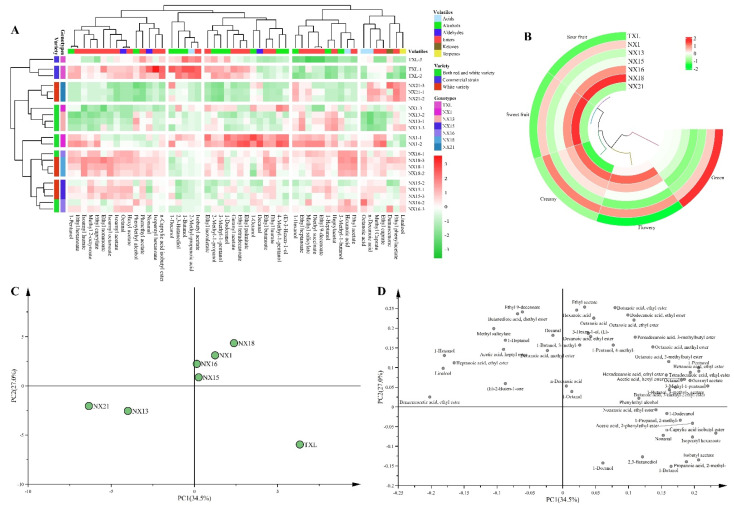
Volatiles profile in Chardonnay wine fermented by 6 indigenous *S. cerevisiae* strains with varying genotypes. (**A**): Heatmap analysis of volatile compounds in Chardonnay wines. (**B**): Aroma attributes of the Chardonnay wines. Principal component analysis (PCA) score (**C**) and loading (**D**) plots of the Chardonnay wines.

The impact of *S. cerevisiae* genotypes on wine volatile compounds was further examined using HS-SPME-GC-MS. A total of 49 volatile compounds were identified and quantified in Cabernet Sauvignon wines, and 50 volatiles for Chardonnay wines (Figure 5A and Figure 6A, Tables S5 and S6). For Cabernet Sauvignon fermentations, 46 out of 49 volatile compounds (except nonanal, geranyl acetate and ethyl tetradecanoate) were significantly different between *S. cerevisiae* genotypes (Table S5). 15 volatiles, including seven esters, five higher alcohols, two fatty acids and one aldehyde had odour activity values (OAV) >1 (Table 2), indicating their direct contribution to wine aroma formation. Although most volatile compounds did not surpass aroma thresholds, their interactions with other compounds may indirectly lead to global changes in wine aroma [56]. Here, we focused on volatiles with concentrations surpassing their sensory thresholds. Among the ethyl esters of medium chain fatty acids with OAV > 1, ethyl hexanoate and ethyl isovalerate, which were documented as contributing to fruity aromas [57], were richer in wines produced by all tested indigenous strains except NX2, NX4 than XR wines (Figure 5A). NX2 and NX4 wines had lower concentrations of ethyl caprate compared to XR wines whilst the highest value of ethyl caprate was observed in NX24 wines (Table 2). Ethyl caprylate and ethyl heptanoate were also 1.4-fold and 1.8-fold higher in NX24 wines than XR wines, respectively (Table 2). Many studies had reported that higher levels of ethyl esters of fatty acids in wine samples followed greater accumulation of the respective fatty acid precursors [55,56]. However, in this study, such correspondence between fatty acids and their ethyl esters was not observed (Table S5). The volatile fatty acid ethyl esters can be synthesised by *S. cerevisiae* with participation of several esterases and ethanol acyltransferases, e.g., ETH1 and EEB1 [57]. It would therefore be interesting to test whether the indigenous yeast strains possess varied enzyme activities that might lead to different levels of fatty acid ethyl ester synthesis. Conversely, a general reduction in acetate esters was found in wines fermented with the indigenous *S. cerevisiae* (Figure 5A). This decrease can be driven by ethyl acetate, a major component of acetate esters with the highest OAV seen in wines (Table 2, Table S5). Only NX16 and NX24 yielded ethyl acetate slightly above 150 mg/L, a level where it is usually considered as a fault rather than providing fruity aromas [57]. All tested yeast strains produced considerable amounts of higher alcohols (Table S5), and their impact on wine aroma modulation depends on the complicated wine matrix [58] and, therefore, needs further investigation. The most abundant higher alcohol in all wines was 3-methyl-1-butanol, followed by phenylethyl alcohol, in agreement with several previous studies [27,28]. The lowest level of 3-methyl-1-butanol and phenylethyl alcohol was detected in NX23 wines, and the highest in NX4 (3-methyl-1-butanol) or NX22 wines (phenylethyl alcohol) wines (Table 2). The 15 compounds with OAV > 1 were further divided into five categories of sensory traits based on their odour characteristics, comprising sour fruit, floral, creamy, sweet fruit and green, and was used to predict their sensory characteristics. The Cabernet Sauvignon wines fermented using 11 indigenous *S. cerevisiae* might exhibit a richer flavour, especially fruity and floral, than those produced with XR (Figure 5B). Of particular interest was the sensory simulation of NX16 wines, showing the strongest sweet fruit, creamy and sour fruit aromas whilst the green aromas were weak (Figure 5B).

**Table 2:** Concentrations (μg/L) of volatile compounds with OAV > 1 in Cabernet Sauvignon wines fermented by the indigenous *S. cerevisiae* strains and the commercial strain XR.

Compounds	XR	NX1	NX2	NX3	NX4	NX5	NX12	NX13	NX16	NX22	NX23	NX24
***Higher alcohols***												
Phenylethyl alcohol	167,392.81 ± 8101.51 b	166,138.4 ± 16,137.44 b	184,165.29 ± 1262.85 b	163,080.39 ± 5833.96 b	181,400.58 ± 13,807.16 b	153,952.72 ± 6061.12 bc	161,268.44 ± 2111.01 b	172,947.88 ± 9876.84 b	170,013.97 ± 5498.8 b	219,079.07 ± 20,367.73 a	122,070.6 ± 4850.72 c	122,826.97 ± 3810.22 c
1-Butanol	4032.25 ± 55.28 c	3255.38 ± 191.63 ghi	3506.21 ± 41.77 efg	3866.75 ± 148.69 cde	4516.09 ± 38.29 b	3612.9 ± 91.64 fg	3327.66 ± 123.64 hi	3014.35 ± 104.99 i	3470.17 ± 63.07 gh	4524.09 ± 77.16 b	2634.42 ± 48.23 j	3126.15 ± 87.79 hi
1-Hexanol	5317.06 ± 25.64 b	4570.32 ± 91.29 fg	4300.47 ± 49.38 ghi	4243.81 ± 55.95 hi	4887.98 ± 9.76 ef	4610.74 ± 60.89 fg	4961.53 ± 11.53 cd	5975.9 ± 185.07 a	4925.24 ± 74.53 de	5258.73 ± 58.39 bc	4358.67 ± 89.23 gh	5012.94 ± 105.5 cd
2,3-Butanediol	111,590.44 ± 3845.3 ef	123,330.57 ± 14,588.11 de	98,891.31 ± 10,380.16 ef	132,756.81 ± 19,232.17 de	90,805.68 ± 7400.75 ef	81,275.53 ± 6401.69 ef	77,136.82 ± 4803.14 ef	57,625.82 ± 1910.63 f	108,578.58 ± 14,651.52 ef	162,414.27 ± 18,866.1 bc	96,817.49 ± 12,192.06 ef	92,397.23 ± 3723.52 ef
3-Methyl-1-butanol	380,702.13 ± 4759.75 de	387,432.53 ± 8767 cd	414,953.11 ± 3126 ab	387,767.4 ± 5091.16 cd	416,610.34 ± 2661.54 a	353,892.33 ± 3751.61 ef	379,568.41 ± 2894.13 de	401,987.72 ± 14,710.3 cd	401,066.72 ± 5778.98 cd	378,320.26 ± 3211.6 de	287,741.45 ± 5179.77 g	305,563.44 ± 6784.64 g
***Esters***												
Ethyl acetate	141,076.1 ± 570.96 b	122,903.61 ± 2959.39 de	121,830.71 ± 783.22 fg	137,144.55 ± 2347.51 b	113,410.61 ± 606.22 gh	115,354.37 ± 1619.63 gh	110,158.64 ± 851.51 h	114,443.65 ± 3603.46 gh	155,122.62 ± 2309.6 a	113,190.06 ± 735.67 gh	135,861.31 ± 2615.16 bc	150,842.72 ± 2602.23 a
Ethyl butanoate	334.9 ± 11.72 ef	259.71 ± 16.41 jk	222.11 ± 3.17 k	305.62 ± 11.79 fg	276.33 ± 5.71 hi	294.94 ± 13.39 gh	342.9 ± 8.56 de	441.1 ± 20.65 b	401.13 ± 14.64 bc	550.72 ± 6.63 a	271.19 ± 7.64 ghij	369.46 ± 10.9 cd
Ethyl caprate	639.06 ± 0.87 cde	552.32 ± 6.67 efghi	533.78 ± 11.62 fghi	523.65 ± 17.35 ghi	470.09 ± 16.86 i	501.33 ± 5.29 hi	497.1 ± 24.02 hi	587.38 ± 74.11 defgh	710.46 ± 16.93 bc	581.51 ± 15.24 defgh	751 ± 44.33 ab	828.31 ± 38.13 a
Ethyl caprylate	2346.69 ± 19.12 cd	2081.53 ± 30.68 de	2101.35 ± 7.97 de	2264.03 ± 66.28 cd	1895.57 ± 25.46 e	2097.47 ± 27.7 de	2250.86 ± 31.97 cde	2325.33 ± 303.39 cd	2771.84 ± 39.05 b	2246.45 ± 36.88 cde	2796.76 ± 97.31 b	3243.46 ± 105.34 a
Ethyl heptanoate	14.8 ± 3.21 def	15.02 ± 4.25 def	13.97 ± 1.13 efg	17.25 ± 1.18 cd	13.15 ± 4.11 fg	16.14 ± 3.41 cde	17.01 ± 2.24 cd	23 ± 2.16 b	17.74 ± 2.08 c	16.96 ± 4.31 cd	24.51 ± 2.65 b	27.16 ± 0.94 a
Ethyl hexanoate	1399.44 ± 8.17 fg	1407.03 ± 41.28 fg	1328.87 ± 24.21 g	1628.04 ± 28.63 de	1341.56 ± 5.21 g	1522.38 ± 40.04 ef	1506.59 ± 15.63 ef	1761.7 ± 69.05 cd	1897.43 ± 22.3 bc	1585.58 ± 31.68 e	1994.14 ± 39.93 ab	2130.84 ± 53.18 a
Ethyl isovalerate	190.24 ± 1.19 cd	190.76 ± 0.31 cd	216.75 ± 10.97 bc	218.99 ± 5.04 bc	242.63 ± 5.71 ab	222.18 ± 8.14 bc	260.02 ± 3.02 a	246.26 ± 11.62 ab	222 ± 16.6 bc	214.27 ± 10.6 bc	150.54 ± 9.56 e	155.04 ± 12.96 de
***Fatty acids***												
Hexanoic acid	1787.69 ± 43.28 f	1959.13 ± 180.21 def	1836.25 ± 31.86 f	2041.96 ± 66.68 cdef	1876.05 ± 97.95 ef	2054.75 ± 102.65 cdef	2194.66 ± 14.1 abcde	2479.7 ± 97.51 a	2102.49 ± 58.76 bcdef	2279.8 ± 126.28 abcd	2251.77 ± 139.77 abcd	2390.67 ± 88.19 ab
Octanoic acid	1308.31 ± 30.95 ef	1376.76 ± 40.1 def	1425.21 ± 18.1 cdef	1384.72 ± 42.99 def	1257.54 ± 77.07 f	1456.28 ± 50.16 bcde	1515.97 ± 3.99 abcd	1601.95 ± 73.22 abc	1369.78 ± 41.93 def	1598.34 ± 99.93 abc	1710.75 ± 72.14 a	1639.71 ± 58.75 ab
***Carbonyl Compounds***												
Octanal	10.09 ± 1.19 ab	3.49 ± 1.8 ab	7.23 ± 0.19 ab	6.17 ± 3.59 ab	3.43 ± 0.33 ab	5.99 ± 0.17 ab	4.85 ± 1.84 ab	11.35 ± 1.09 ab	2.06 ± 0.56 b	3.79 ± 2 ab	9.37 ± 3.57 ab	4.16 ± 2.33 ab

Data are presented as mean values of triplicates ± standard deviation (μg/L). Values within the same row followed by different letters are significantly different (*p* < 0.05).

In Chardonnay wines, of the 50 volatiles identified and quantified, 47 showed significant difference (Table S6), among which concentrations of 13 compounds were beyond their sensory thresholds (Table 3). Again, the indigenous *S. cerevisiae* population dedicated to more complex volatile compositions compared to the commercial reference strain TXL (Figure 6A). NX1 was inclined to yield a wide range of branched-chain alcohols whilst NX1, NX15, NX16 and NX18 generated higher amounts of pleophyletic esters (Figure 6A). These esters, described as contributing full-bodied fruity aromas, were commonly provided by non-*Saccharomyces* yeasts [46]. Our findings highlight the potential of indigenous *S. cerevisiae* in enhancing aromatic ester production that would attribute to the desirable fruity and floral aromas, especially NX16 and NX18 (Figure 6A,B). In contrast, the commercial strain TXL might enhance the green characters of the Chardonnay wines (Figure 6B). Interestingly, the volatile profiles of NX1, and NX16 Chardonnay wines display inconsistency with the corresponding Cabernet Sauvignon wines (Figure 5A and Figure 6A), which is likely due to the interactions of *S. cerevisiae* strains and the grape variety. For example, many fermentation-derived higher alcohols are produced from the corresponding amino acid metabolism through the Ehrlich pathway [58]. Factors like preferential consumption of amino acids between *S. cerevisiae* strains and the amino acid composition of grape juice/must would clearly affect the higher alcohol formation, and subsequently exert influences on the corresponding esters.

To visualize the relationship between *S. cerevisiae* genotypes with all volatile compounds of Cabernet Sauvignon and Chardonnay wines, principal component analysis was conducted. The first three/two principal components (PCs) allowed clear separation of Cabernet Sauvignon and Chardonnay wines fermented by *S. cerevisiae* with different genotypes (Figure 5C,D and Figure 6C,D). For Cabernet Sauvignon wines, the first three PCs accounted for 65.6% of the variation in volatile compounds. Separation of PC1 was driven by a number of ethyl esters (e.g., ethyl phenylacetate and ethyl isovalerate) and some higher alcohols (e.g., 1-octanol, 1-decanol and 1-pentanol) (Figure 5C), with NX24, NX23, NX13, NX16 and XR located on the negative axis of the plot, and the rest of the wines located on the positive axis of the plot (Figure 5C). Phenethyl acetate and 3-methyl-1-butanol acetate drove the separation of PC2 towards the right hand-side of the plot, co-localising with NX16 wines, whilst ethyl phenylacetate and ethyl 9-decenoate drove the separation of PC2 towards the left hand-side of the plot (Figure 5C,D). Several higher alcohols such as 4-methyl-1-pentanol, 3-methyl-1-pentanol, 1-hexanol, and ethyl isovalerate drove the separation of PC3 towards the bottom of the plot (Figure 5D), co-localising with NX13 wines (Figure 5C).

For Chardonnay wines, the first two PCs accounted for 61.5% of the variation in volatile compounds (Figure 6C). A clear separation according to different indigenous *S. cerevisiae* was seen on PC1, which explained 34.5% of the overall variation. Among the tested *S. cerevisiae*, NX1, NX15, NX16, NX18 and TXL wines were located at the positive axis of PC1, and were associated with several ethyl esters of fatty acids (e.g., ethyl caprate), acetate esters (e.g., geranyl acetate) and fatty acids (e.g., n-decanoic acid) (Figure 6C,D) whilst NX13 and NX21 wines were on the bottom left quadrant of the plot (Figure 6C,D). PC2, accounted for 27.0% of variation, further separating NX1, NX15, NX16 and NX18 wines from the wines fermented by the commercial strain TXL (Figure 6C). TXL wines were located towards the bottom of PC2, and were primarily affected by higher production of 1-butanol, 1-decanol, 2,3-butanediol, 2-methyl-propanoic acid, and isobutyl acetate (Figure 6D).

## 4. Conclusions

In conclusion, this study describes the isolation, identification and Interdelta genotyping of indigenous yeasts from spontaneous fermentations (with/without SO_2_ addition) using eight typical grape varieties in a single vineyard. The representative *S. cerevisiae* genotypes were then evaluated for their vinification characteristics. Our findings highlighted the interactions of grape varieties and supplementation with SO_2_ on shaping yeast diversity, in particular *S. cerevisiae* populations during fermentation. We also observed evident differentiation of *S. cerevisiae* populations at vineyard scale, and the genetic divergence of *S. cerevisiae* strongly influenced the physiochemical parameters and volatile profiles of the wine. Several indigenous *S. cerevisiae* strains (e.g., NX16 and NX18) have potential for industrial use as starter cultures based on their excellent capacity to form desirable volatile compounds. Further studies are under way to evaluate their oenological-associated characteristics as well as investigating their vinification profiles at winery-scale fermentation.

## Figures and Tables

**Table 1:** Distribution of 33 *S. cerevisiae* genotypes during spontaneous fermentations with eight typical grape varieties. Numbers outside the brackets represent the number of the genotype found at each stage of fermentation whilst numbers in the brackets show the percentage of the specific genotype.

Type	Riesling				Semillon				Cabernet Sauvignon				Cabernet Gernischet					Cinsault				Pinot Noir			Yan73			Merlot		
	NS		S		NS		S		NS		S		NS		S			NS		S		NS		S	NS		S	NS	S	
	M	E	M	E	M	E	M	E	M	E	M	E	M	E	I	M	E	M	E	M	E	M	E	E	M	E	E	E	M	E
NX1	3 (38)	8 (62)	7 (88)	7	1 (17)	2 (17)	1(14)	5(46)	-	-	-	-	-	-		-	-	-	-	5	13	-	-	-	1	-	-	17 (65)	1	16 (88)
NX2	-	-	-	-	-	-	-	-	1 (12)	2 (17)	2 (14)	1 (7)	-	-		-	-	-	-	-	-	-	-	-	-	-	-	-	-	-
NX3	-	-	-	-	-	-	-	-	-	-	-	-	-	-		7 (50)	6 (55)	-	-	-	-	-	-	-	-	-	-	-	-	-
NX4	-	-	-	-	-	-	-	-	7 (88)	7 (58)	11 (79)	10 (72)	7 (70)	7 (50)	3 (75)	7 (50)	3 (27)	-	-	-	-	-	-	-	-	-	-	-	-	-
NX5	-	-	-	-	-	-	-	-	-	-	-	-	1 (10)	5 (36)	-	-	-	-	-	-	-	-	-	-	-	-	-	-	-	-
NX6	-	-	-	-	-	-	-	-	-	-	-	-	-	1 (7)	-	-	-	-	-	-	-	-	-	-	-	-	-	-	-	-
NX7	-	-	-	-	-	-	-	-	-	-	-	-	-	-	-	-	1 (9)	-	-	-	-	-	-	-	-	-	-	-	-	-
NX8	-	-	-	-	-	-	-	-	-	-	-	-	2 (20)	-	-	-	-	-	-	-	-	-	-	-	-	-	-	-	-	-
NX9	-	-	-	-	-	-	-	-	-	-	-	-	-	1 (7)	-	-	-	-	-	-	-	-	-	-	-	-	-	-	-	-
NX10	-	-	1 (12)	-	-	-	-	-	-	-	-	-	-	-	-	-	-	-	-	-	-	-	-	-	-	-	-	-	-	-
NX11	-	-	-	-	-	1 (8)	-	-	-	-	-	-	-	-	-	-	-	-	-	-	-	-	-	-	-	-	-	-	-	-
NX12	-	-	-	-	-	-	-	-	-	2 (17)	1 (7)	2 (14)	-	-	-	-	-	-	-	-	-	-	-	-	-	-	-	-	-	-
NX13	5 (62)	4 (30)	-	-	-	-	-	-	-	-	-	-	-	-	-	-	-	-	-	-	-	-	-	-	-	11 (84)	11 (92)	-	-	-
NX14	-	-	-	-	-	-	1 (14)	-	-	-	-	-	-	-	-	-	-	-	-	-	-	-	-	-	-	-	-	-	-	-
NX15	-	1 (8)	-	-	-	-	-	-	-	-	-	-	-	-	-	-	-	-	-	-	-	-	-	-	-	-	-	-	-	-
NX16	-	-	-	-	5 (83)	7 (58)	4 (58)	2	-	-	-	-	-	-	-	-	-	1	2(50)	-	-	-	-	-	-	-	-	-	-	-
NX17	-	-	-	-	-	1 (8)	-	-	-	-	-	-	-	-	-	-	-	-	-	-	-	-	-	-	-	-	-	-	-	-
NX18	-	-	-	-	-	-	-	4	-	-	-	-	-	-	-	-	-	-	-	-	-	-	-	-	-	-	-	-	-	-
NX19	-	-	-	-	-	1 (8)	-	-	-	-	-	-	-	-	-	-	-	-	-	-	-	-	-	-	-	-	-	-	-	-
NX20	-	-	-	-	-	-	-	-	-	-	-	-	-	-	1 (25)	-	1 (9)	-	-	-	-	-	-	-	-	-	-	-	-	-
NX21	-	-	-	-	-	-	1 (14)	-	-	-	-	-	-	-	-	-	-	-	-	-	-	-	-	-	-	-	-	-	-	-
NX22	-	-	-	-	-	-	-	-	-	1 (8)	-	1 (7)	-	-	-	-	-	-	-	-	-	-	-	-	-	-	-	-	-	-
NX23	-	-	-	-	-	-	-	-	-	-	-	-	-	-	-	-	-	-	-	-	-	1 (50)	11 (92)	9	-	-	-	-	-	-
NX24	-	-	-	-	-	-	-	-	-	-	-	-	-	-	-	-	-	-	-	-	-	1 (50)	-	-	-	-	-	-	-	-
NX25	-	-	-	-	-	-	-	-	-	-	-	-	-	-	-	-	-	-	-	-	-	-	1 (8)	-	-	-	-	-	-	-
NX26	-	-	-	-	-	-	-	-	-	-	-	-	-	-	-	-	-	-	-	-	-	-	-	-	-	1 (8)	-	-	-	-
NX27	-	-	-	-	-	-	-	-	-	-	-	-	-	-	-	-	-	-	-	-	-	-	-	-	-	1 (8)	1 (8)	-	-	-
NX28	-	-	-	-	-	-	-	-	-	-	-	-	-	-	-	-	-	-	2 (50)	-	-	-	-	-	-	-	-	-	-	-
NX29	-	-	-	-	-	-	-	-	-	-	-	-	-	-	-	-	-	-	-	-	-	-	-	-	-	-	-	2 (8)	-	-
NX30	-	-	-	-	-	-	-	-	-	-	-	-	-	-	-	-	-	-	-	-	-	-	-	-	-	-	-	2 (8)	-	-
NX31	-	-	-	-	-	-	-	-	-	-	-	-	-	-	-	-	-	-	-	-	-	-	-	-	-	-	-	3 (11)	-	1 (6)
NX32	-	-	-	-	-	-	-	-	-	-	-	-	-	-	-	-	-	-	-	-	-	-	-	-	-	-	-	2 (8)	-	-
NX33	-	-	-	-	-	-	-	-	-	-	-	-	-	-	-	-	-	-	-	-	-	-	-	-	-	-	-	-	-	1 (6)
Total	8	13	8	7	6	12	7	11	8	12	14	14	10	14	4	14	11	1	4	5	13	2	12	9	1	13	12	26	1	18
		290																												

Short string referred to ‘no detected’. NS, SO_2_-free groups; S, SO_2_ treated groups; I, initial stage of fermentation; M, middle stage of fermentation; E, end stage of fermentation. Green filling represents the special genotypes of *Saccharomyces* observed during spontaneous fermentations without SO_2_. Orange filling represents the special genotypes of *Saccharomyces* observed during spontaneous fermentations with SO_2_.

**Table 3:** Concentrations (μg/L) of volatile compounds with OAV > 1 in Chardonnay fermented by the indigenous *S. cerevisiae* strains and the commercial strain TXL.

Compounds	TXL	NX1	NX13	NX15	NX16	NX18	NX21
***Higher alcohols***							
1-Hexanol	1971.76 ± 147.92 f	2842.83 ± 146.66 ab	2651.52 ± 74.29 abc	2309.15 ± 72.57 e	2628.71 ± 85.34 abcd	2570.46 ± 37.14 bcde	2918.71 ± 13.84 a
1-Butanol	2226.23 ± 191.1 a	1294.55 ± 193.17 b	878.94 ± 74.01 cde	788.7 ± 53.8 e	994.74 ± 26.87 bcde	1129.32 ± 15.8 bcd	1099 ± 48.95 bcde
Phenylethyl alcohol	33,452.18 ± 217.87 bc	30,548.64 ± 920.91 bcde	34,624.57 ± 859.49 b	31,241.83 ± 746.26 bcd	34,075.19 ± 1382.96 b	34,578.42 ± 73.79 b	27,445.21 ± 781.72 def
2,3-Butanediol	62,208.64 ± 7896.55 a	52,996.87 ± 6709.23 abc	42,002.45 ± 2192.48 abcd	29,209.82 ± 1752.84 d	44,376.28 ± 6013.81 abcd	37,672.03 ± 1337.7 bcd	41,197.97 ± 876.81 abcd
***Esters***							
Ethyl butanoate	1437.82 ± 51.97 de	1909.82 ± 107.85 b	1540.11 ± 56.39 cd	1751.26 ± 72.09 bc	1802.22 ± 100.98 b	1965.09 ± 67.34 ab	1384.46 ± 4.52 de
Ethyl heptanoate	2.22 ± 0.2 h	5.93 ± 0.59 ab	6.34 ± 0.29 a	4.17 ± 0.11 cde	4.65 ± 0.41 cd	4.38 ± 0.24 cd	4.91 ± 0.02 bc
Ethyl hexanoate	3314.12 ± 207.96 ab	3309.02 ± 261.28 ab	2450.15 ± 113.84 e	3044.31 ± 115.22 bcd	3109.39 ± 214.3 bc	3236.18 ± 139.05 b	2705.37 ± 0.34 cde
Ethyl acetate	124,257.25 ± 5737.59 efgh	154,587.96 ± 11,783.89 bcd	137,846.7 ± 4915.97 de	144,759.19 ± 5168.89 cde	163,631.1 ± 7155.4 bc	175,981.76 ± 4627.54 ab	132,947.74 ± 934.09 defg
Ethyl caprylate	5949.03 ± 668.51 bcd	6576.43 ± 951.39 bc	5287.53 ± 169.86 cde	6646.79 ± 157.89 bc	6695.31 ± 402.05 bc	7301.32 ± 195.72 ab	5847.88 ± 10.79 bcd
Ethyl caprate	2509.32 ± 368.15 bcde	3258.82 ± 550.69 ab	2031.23 ± 42.4 de	2915.93 ± 86.19 abcd	2761.74 ± 167.02 abcd	3141.74 ± 15.44 ab	2951.21 ± 162.31 abc
***Fatty acids***							
Octanoic acid	5089.21 ± 373.88 fghi	5782.67 ± 160.38 cdefg	4734.89 ± 72.96 hi	5864.91 ± 159.48 bcdef	6352.34 ± 414.9 abcd	6214.38 ± 93.05 abcde	5480.99 ± 169.41 efghi
Hexanoic acid	3699.21 ± 17.38 e	4526.67 ± 195.94 bc	4003.64 ± 158.99 de	4202.46 ± 160.85 cd	5018.69 ± 100.03 a	5016.69 ± 69.15 a	4035 ± 85.38 de
***Carbonyl Compounds***							
Octanal	315.83 ± 16.9 b	257.69 ± 22.57 c	240.71 ± 9.58 c	324.74 ± 10.24 b	321.02 ± 19.74 b	322.93 ± 11.01 b	226.68 ± 0.17 cd

Data are presented as mean values of triplicates ± standard deviation (μg/L). Values within the same row followed by different letters are significantly different (*p* < 0.05).

## Data Availability

Not applicable.

## Supplementary Materials

The following supporting information can be downloaded at: https://www.mdpi.com/article/10.3390/microorganisms10071455/s1, Figure S1: Interdelta fingerprinting patterns of the 290 indigenous *S. cerevisiae* isolates. Table S1: Dynamics of yeast population during spontaneous fermentations. Table S2: 712 isolates obtained from spontaneous fermentations and species identification by WL nutrient medium and sequencing of the 26s rRNA D1/D2 domain. Table S3: Categories of morphotypes of yeast strains isolated from grape spontaneous fermentation. Table S4: Colony numbers and percentage (%) of *Saccharomyces* isolates and genotypes during wine spontaneous fermentations with/without SO_2_ addition. Table S5: Concentration (μg/L) of volatile compounds in Cabernet Sauvignon wines fermented by indigenous *S. cerevisiae* with varying genotypes. Table S6: Concentration (μg/L) of volatile compounds in Chardonnay wines fermented by indigenous *S. cerevisiae* with varying genotypes.
