# Supplementary material for: Yeast Diversity during Spontaneous Fermentations and Oenological Characterisation of Indigenous Saccharomyces cerevisiae for Potential as Wine Starter Cultures
Source: Microorganisms. 2022 Jul 19;10(7):1455. doi: 10.3390/microorganisms10071455 (PMC9325129; doi:10.3390/microorganisms10071455)
Supplement: Supplementary file 1 [file microorganisms-10-01455-s001.zip › Supplementary figure.pdf]

Fermentations and Oenological Characterisation of Indigenous Saccharomyces cerevisiae for Potential as Wine Starter Cultures

Yu Chen [1,†], Jiao Jiang [1,2,†], Yaoyao Song [1], Xiaomin Zang [1], Guoping Wang [1], Yingfang Pei [1], Yuyang Song [1,2,3], Yi Qin [1,2,3,*] and Yanlin Liu [1,2,3,*]

[1] College of Enology, Northwest A & F University, Yangling 712100, China

[2] Ningxia Helan Mountain's East Foothill Wine Experiment and Demonstration Station of Northwest A&F University, Yongning 750104, China

[3] Shaanxi Engineering Research Center for Viti-Viniculture, Yangling 712100, China

[*] Correspondence: yanlinliu@nwsuaf.edu.cn (YL. Liu); qinyi@nwsuaf.edu.cn (Y. Qin)

Figure S1. The gel electrophoresis of Interdelta fingerprinting patterns from 290 isolates.

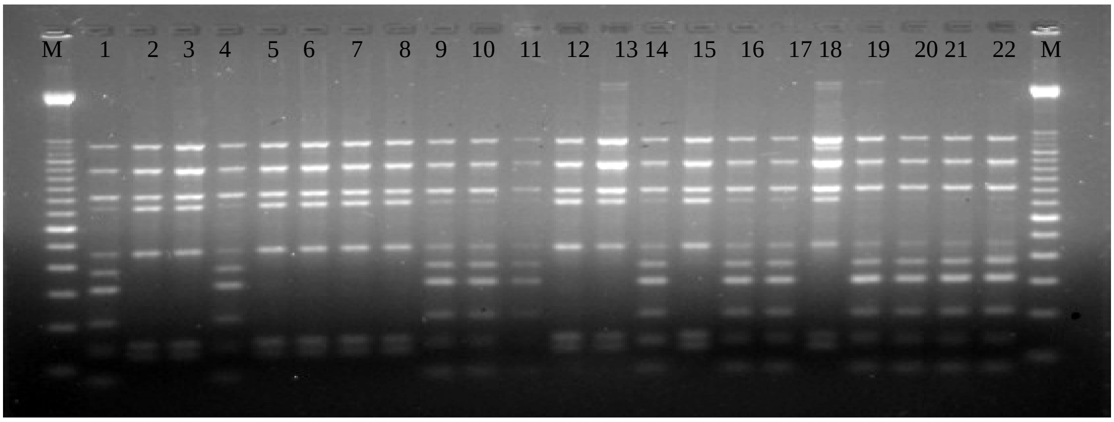

Figure S1-A

M: Marker, 1: E2-2, 2: E2-4, 3: E2-8, 4: E2-9, 5: E2-10, 6: E2-11, 7: E2-12, 8: E3-1, 9: E3-2, 10: E3-3, 11: E3-4, 12: E3-5, 13: E3-9, 14: E3-10, 15: E3-13, 16: E3-12, 17: E3-13, 18: E3-15, 19: ES2-2, 20: E2-3, 21: ES2-12, 22: ES2-13

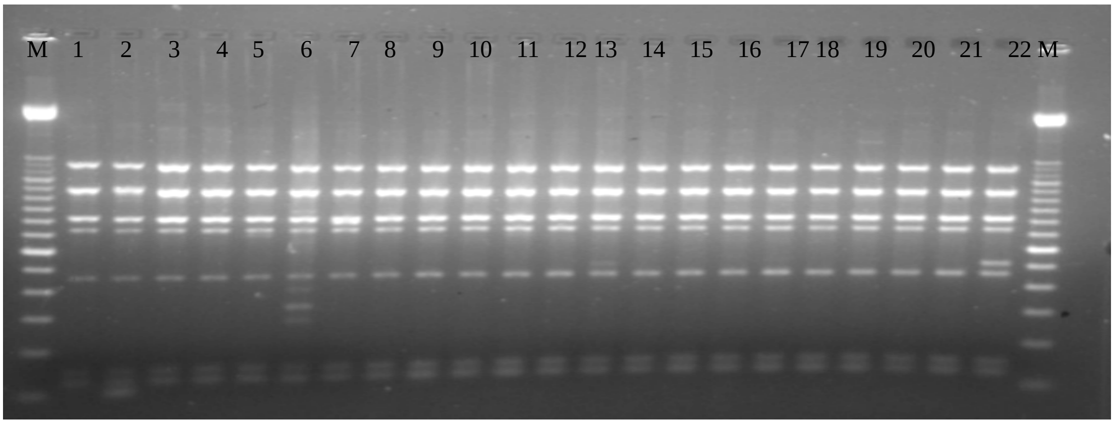

Figure S1-B

M: Marker, 1: I3-1, 2: I3-2, 3: I3-3, 4: I3-4, 5: I3-6, 6: I3-7, 7: I3-8, 8: I3-9, 9: I3-11, 10: I3-12, 11: I3-13, 12: I3-14, 13: I3-15, 14: IS3-2, 15: IS3-3, 16: IS3-4, 17: IS3-5, 18: IS3-6, 19: IS3-7, 20: IS3-8, 21: IS3-9, 22: IS3-10

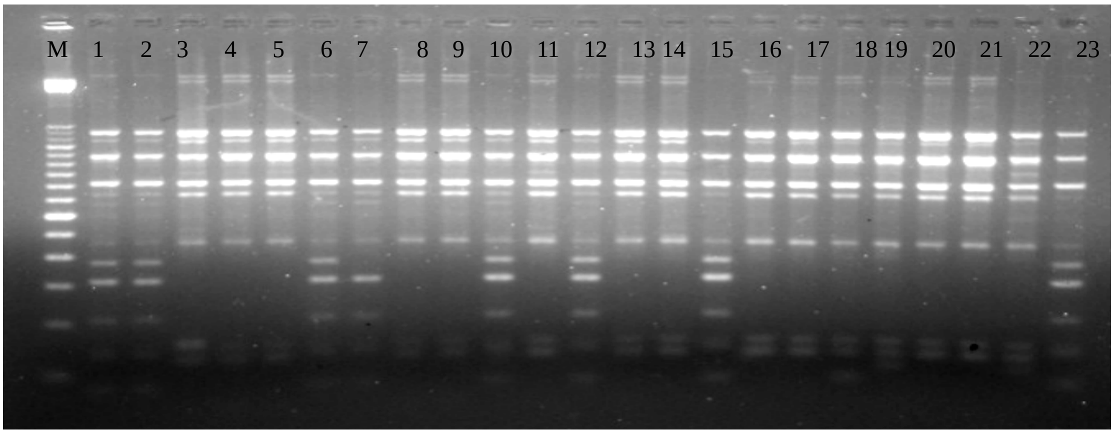

Figure S1-C

M: Marker, 1: FS3-12, 2: FS2-3, 3: FS2-9, 4: FS2-11, 5: FS2-15, 6: F3-2, 7: F3-3, 8: F3-13, 9: F3-

15, 10: FS3-5, 11: FS3-6, 12: FS3-9, 13: FS2-13, 14: FS3-15, 15: F2-3, 16: F2-4, 17: F2-8, 18: F2-9, 19: F2-10, 20: F2-11, 21: F3-1, 22: F3-4, 23: F3-5

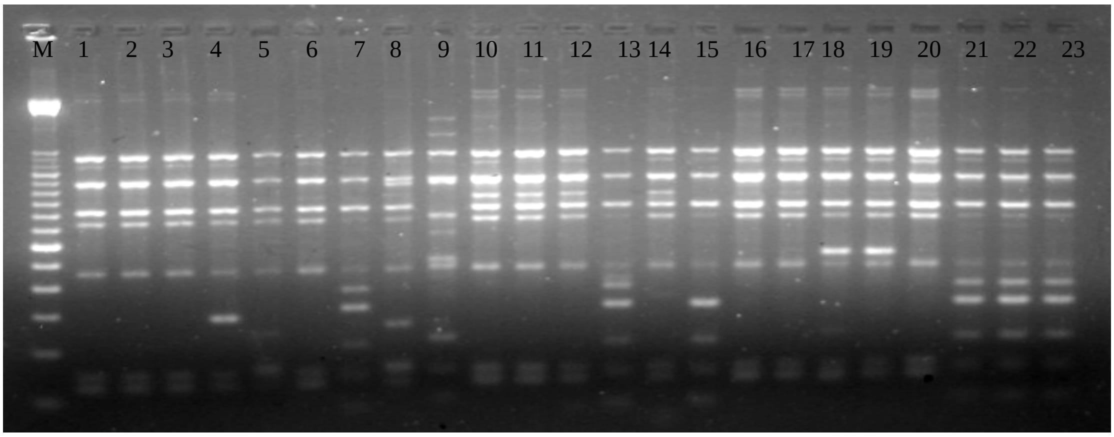

Figure S1-D

M: Marker, 1: F3-6, 2: F3-7, 3: F3-9, 4: F3-10, 5: F3-11, 6: F3-12, 7: FS2-3, 8: FS2-5, 9: FS2-8, 10: FS3-1, 11: FS3-2, 12: FS3-4, 13: FS3-14, 14: FS2-13, 15: FS3-8, 16: B2-5, 17: B3-1, 18: B3-2, 19: B3-13, 20: B3-15, 21: BS2-2, 22: BS2-5, 23: BS2-6

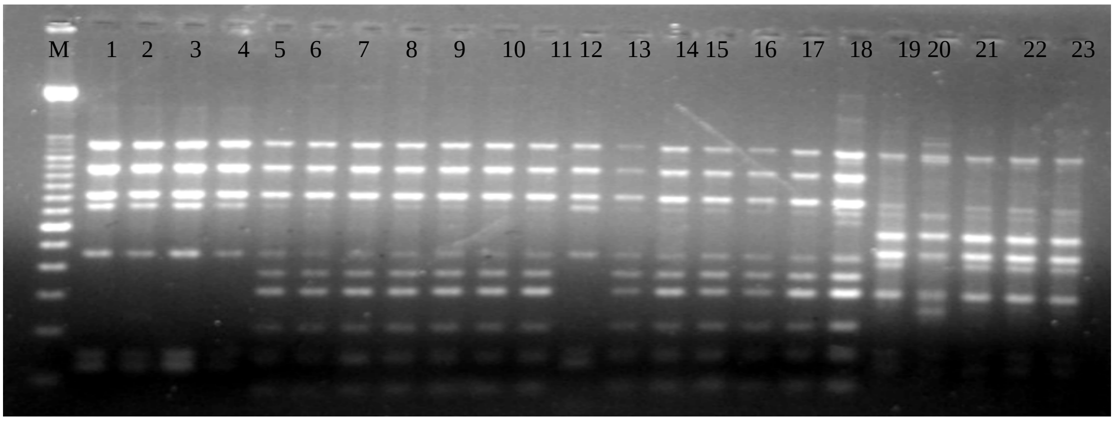

Figure S1-E

M: Marker, 1: IS3-11, 2: IS3-12, 3: IS3-13, 4: I2-3, 5: E2-14, 6: E3-8, 7: ES3-2, 8: ES3-3, 9: ES3-4, 10: ES3-5, 11: ES3-6, 12: E3-14, 13: ES2-8, 14: ES2-16, 15: ES3-1, 16: ES3-7, 17: ES2-6, 18: ES2-5, 19: C3-1, 20: C3-2, 21: C3-4, 22: C3-5, 23: CS3-11

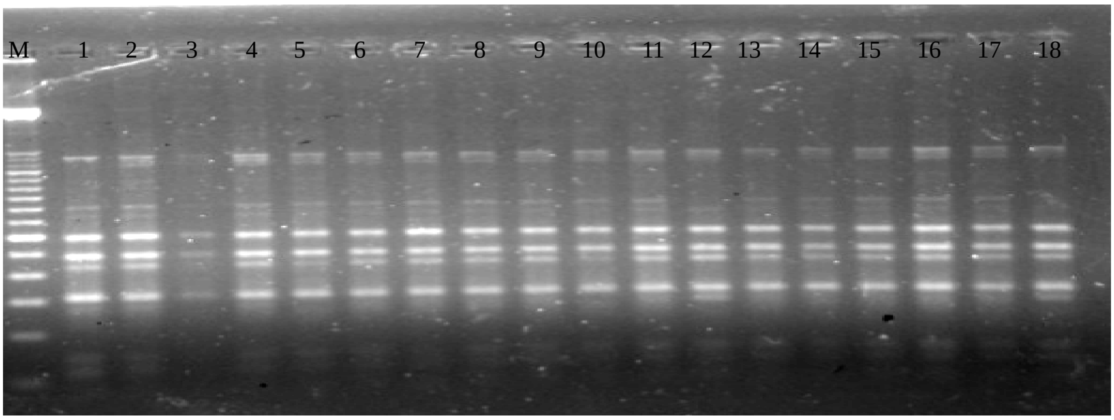

Figure S1-F

M: Marker, 1: CS3-13, 2: C2-3, 3: C2-10, 4: C3-3, 5: CS3-4, 6: C3-6, 7: C3-8, 8: C3-9, 9: C3-10, 10: C3-11, 11: C3-13, 12: CS3-1, 13: CS3-2, 14: CS3-3, 15: CS3-5, 16: CS3-8, 17: CS3-9, 18: CS3-12

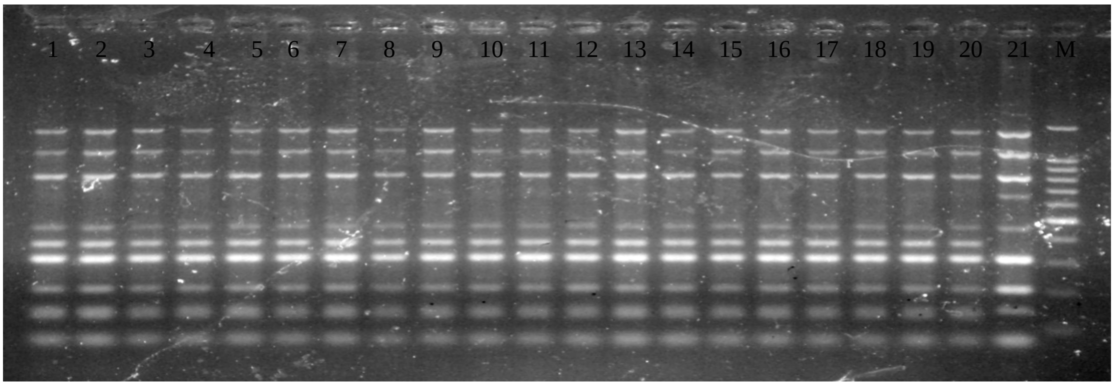

Figure S1-G

M: Marker, 1: BS2-10, 2: BS2-14, 3: BS3-1, 4: BS3-2, 5: BS3-3, 6: BS3-4, 7: BS3-5, 8: BS3-7, 9: BS3-8, 10: BS3-9, 11: BS3-10, 12: BS3-11, 13: BS3-13, 14: BS3-14, 15: BS3-15, 16: HS3-1, 17: HS3-2, 18: HS3-4, 19: HS3-5, 20: HS3-6, 21:JS3-137

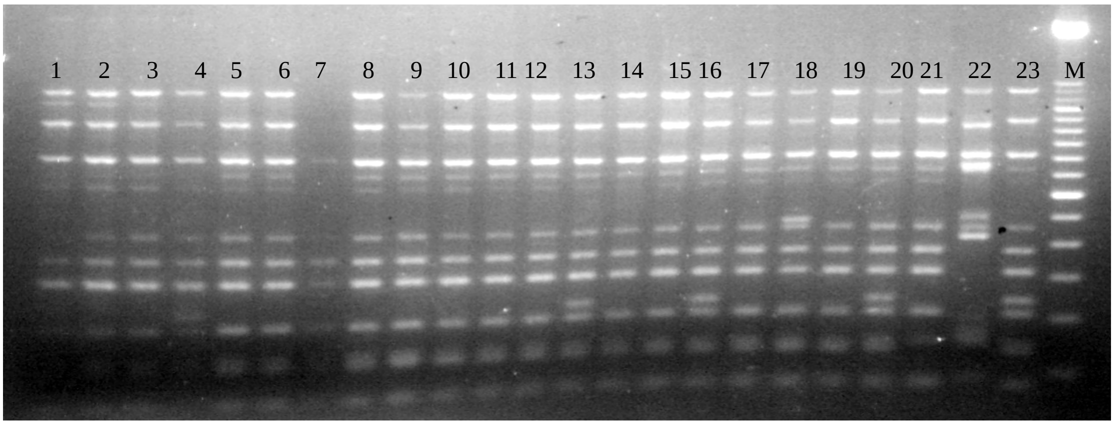

Figure S1-H

M: Marker, 1: A2-1, 2: A2-2, 3: A2-3, 4: A2-5, 5: A2-6, 6: A2-8, 7: A2-9, 8: A2-10, 9: A2-11, 10: A2-12, 11: A3-1, 12: A3-2, 13: A3-3, 14: A3-4, 15: A3-5, 16: A3-6, 17: A3-7, 18: A3-8, 19: A3-9, 20: A3-10, 21: A3-12, 22: A3-13, 23: AS3-148

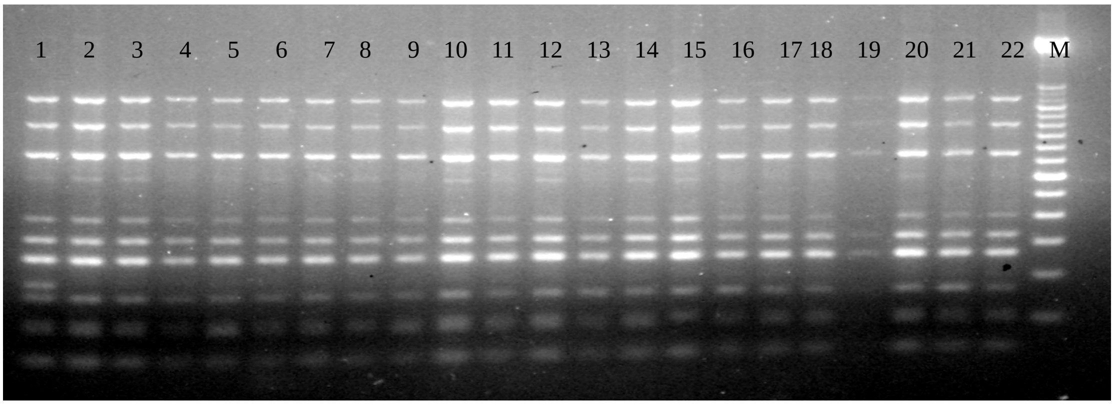

Figure S1-I

M: Marker, 1: AS3-15, 2: AS2-1, 3: AS2-2, 4: AS2-3, 5: AS2-4, 6: AS2-5, 7: AS2-6, 8: AS2-7, 9:

AS2-8, 10: AS2-9, 11: AS2-10, 12: AS2-11, 13: AS2-12, 14: AS2-13, 15: AS2-14, 16: AS3-1, 17: AS3-2, 18: AS3-3, 19: AS3-5, 20: AS3-6, 21: AS3-7, 22: AS3-8

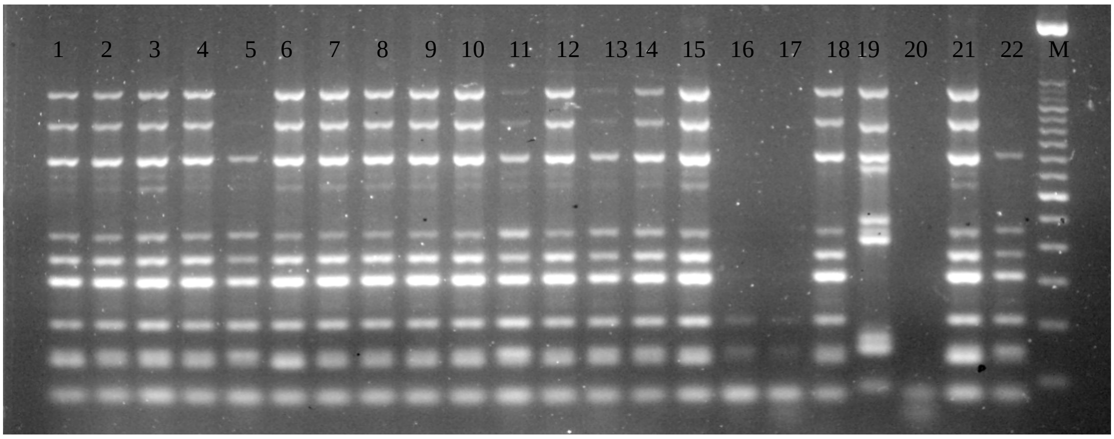

Figure S1-J

M: Marker, 1: J2-3, 2: J2-5, 3: J2-6, 4: J2-8, 5: J2-9, 6: J2-11, 7: J2-12, 8: J3-1, 9: J3-2, 10: J3-3, 11: J3-4, 12: J3-5, 13: J3-6, 14: J3-7, 15: J3-8, 16: J3-9, 17: J3-10, 18: J3-11, 19: J3-12, 20: JS2-1, 21: JS2-2, 22: JS2-3

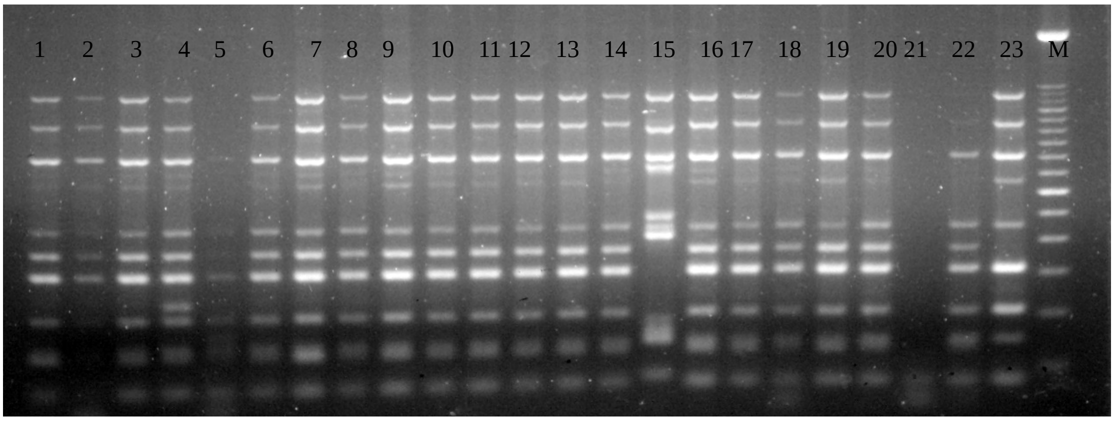

Figure S1-K

M: Marker, 1: JS2-4, 2: JS2-5, 3: JS2-6, 4: JS2-7, 5: JS2-8, 6: JS2-9, 7: JS2-10, 8: JS2-12, 9: JS2-13, 10: JS2-14, 11: JS3-15, 12: JS3-1, 13: JS3-2, 14: JS3-3, 15: JS3-4, 16: JS3-5, 17: JS3-6, 18: JS3-7, 19: JS3-8, 20: JS3-9, 21: JS3-10, 22: JS3-11, 23: JS3-12

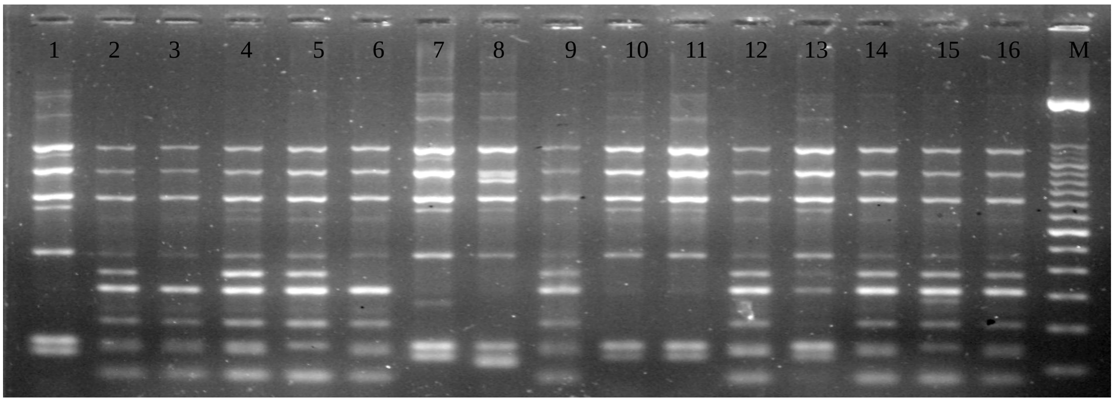

Figure S1-L

M: Marker, 1: G3-1, 2: G3-2, 3: G3-3, 4: G3-4, 5: G3-5, 6: G3-9, 7: G3-10, 8: G3-11, 9: G3-12, 10:

G3-13, 11: G3-14, 12: GS3-1, 13: GS3-2, 14: GS3-3, 15: GS3-4, 16: GS3-5

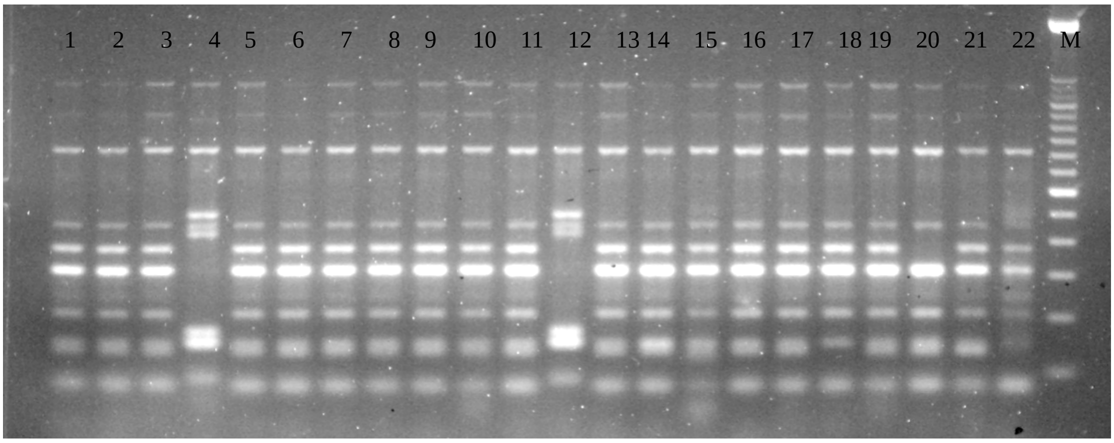

Figure S1-M

M: Marker, 1: D3-1, 2: D3-2, 3: D3-3, 4: D3-4, 5: D3-5, 6: D3-6, 7: D3-7, 8: D3-8, 9: D3-9, 10: D3-10, 11: D3-11, 12: D3-12, 13: D3-13, 14: D3-14, 15: D3-15, 16: DS3-1, 17: DS3-2, 18: DS3-3, 19: DS3-4, 20: DS3-5, 21: DS2-11, 22: J2-1

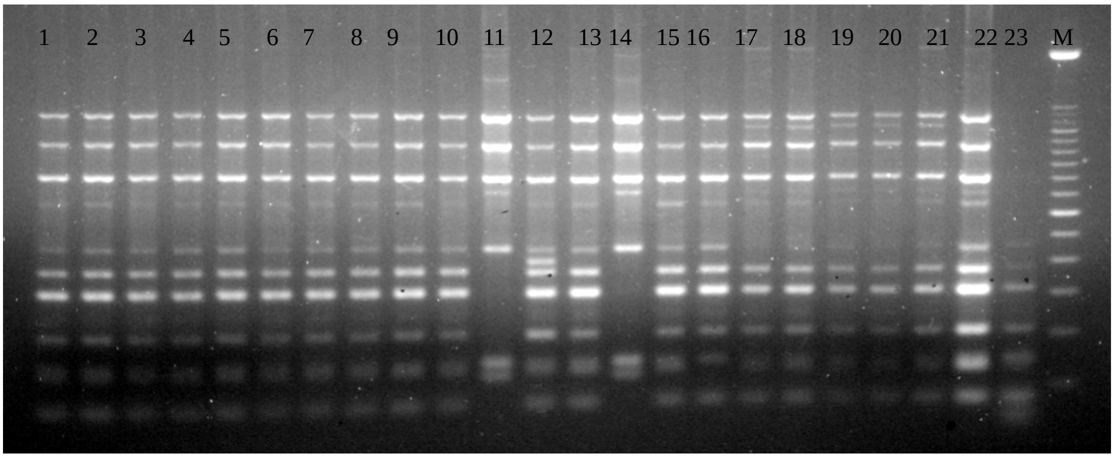

Figure S1-N

M: Marker, 1: GS3-6, 2: GS3-7, 3: GS3-8, 4: GS3-9, 5: GS3-10, 6: GS3-12, 7: GS3-13, 8: GS3-14, 9: AS3-4, 10: AS3-10, 11: AS3-12, 12: AS3-13, 13: AS1-2, 14: AS1-6, 15: AS1-9, 16: AS1-12, 17: HS3-7, 18: HS3-9, 19: HS3-10, 20: HS3-11, 21: HS3-12, 22: JS3-15, 23: JS3-14
